# Supplementary material for: Are carbon emissions trading and green financial instruments synergistic? -Comprehensive quantitative research based on content analysis
Source: PLoS One. 2024 Mar 7;19(3):e0298601. doi: 10.1371/journal.pone.0298601 (PMC10919601; doi:10.1371/journal.pone.0298601)
Supplement: S1 Data — (ZIP) [file pone.0298601.s001.zip › Data/地级市Z_F工作报告.zip!/║╙▒▒/└╚╖╗╩╨/└┤╫╘╣1⁄2╓┌║┼í╛┬φ┐╦╩2╛▌═°í┐_2019.docx]

2018年，我们坚持以习近平新时代中国特色社会主义思想和党的十九大精神为指导，坚决贯彻中央和省各项决策部署，在市委坚强领导下，坚持稳中求进工作总基调，坚持以新发展理念引领高质量发展，积极抢抓京津冀协同发展重大机遇，攻坚克难、拼搏竞进，经济社会发展取得新成就。

一、二○一八年工作回顾

(一)主要指标平稳增长，经济质量进一步优化。预计全市地区生产总值同比增长6.8%，固定资产投资同比增长7%左右，规模以上工业增加值同比增长4%左右，社会消费品零售总额同比增长10%左右，城乡居民人均可支配收入分别增长7.5%和8.8%；一般公共预算收入完成362亿元，同比增长9.2%。预计规模以上工业利润增长25%，同比提高41个百分点；服务业增加值增长11%，对经济增长的贡献率达到70%，三次产业结构由6.5:43.8:49.7调整为6:39:55；单位生产总值能耗下降3.5%以上；研发经费支出占生产总值比重达到1.43%。

(二)深入推动协同发展，一体化步伐进一步加快。对标对表“国家大事”，全面开展北中南三大板块总体规划及系列子规划编制工作，北三县与通州区协同发展“1+1+1+4+8”规划体系、大兴国际机场临空经济区廊坊片区“1+1+1+8”规划体系已取得初步成果，雄安新区周边县(市)城乡总体规划编制工作进展顺利。坚决落实国家和省对京冀交界地区和雄安新区周边地区管控政策，北三县按照与通州区“统一规划、统一政策、统一标准、统一管控”要求，实施贴边区违法建设专项整治行动，对贴边区内项目列出清单、建立台账、分类整治，纳入拆迁范围的33项违法建筑彻底“清零”；全力支持大兴国际机场建设，涉及我市11项配套迁改项目基本完成，回迁区安置房加快建设，噪声区5个村街拆迁和降噪区17个村街降噪工作基本完成；及时出台服务支持雄安新区规划建设的决定和实施方案。加快推动交通、产业、生态环保三大重点领域率先突破，密涿高速廊坊段实现通连，京雄城际铁路、新机场北线高速、津石高速开工建设；中国移动大数据中心、中航试金石检测基地等一批京津转移项目加快建设或建成投产。全年引进京津项目161个、资金660亿元。扎实推进蓝天、碧水、净土三大保卫战，持续强化大气污染综合治理，全面完成7.7万户“煤替代”扫尾工程，三年累计完成99万户，在全省率先实现农村地区散煤“清零”；33台35蒸吨及以下燃煤锅炉淘汰任务全部完成，主城区实现超低排放集中供热全覆盖。动态排查整治“散乱污”企业1788家，深度治理VOCs 企业128家。推广新能源标准车4819辆，完成省定任务的268%。主城区121家在建工地全部安装PM10在线监测设备。在工程停工、错峰生产等10个方面实施差异化监管，禁止“一刀切”。2018年全市空气质量综合指数5.94、同比下降10.14%，在169个国控重点城市中倒排第39位，PM2.5平均浓度下降到52微克/立方米，为近年来最好水平，重污染天数12天、同比减少13天，优良天数222天、同比增加8天，空气质量持续改善，蓝天白云越来越多。着力推进水污染防治，全年新建12座城镇污水处理厂和8座园区污水处理厂，新增污水处理能力28.6万吨，其中5座达到京B标准。全市180条河渠纳入河长制管理。6条国省控重点河流7个考核断面中，5个消除劣Ⅴ类水质，超额完成国省下达年度目标任务。全年营造林107.89万亩，森林覆盖率达到32.22%，涌现出“千亩樱花海”“万亩银杏林”等一批高水平景观项目。

(三)扎实推进项目建设，产业结构进一步优化。实施亿元以上项目1004项，其中10亿元以上项目163项，亿元以上产业项目中战略性新兴产业和现代服务业项目占比达到60%以上；争列省重点项目38项，完成投资占年计划的143.4%。制定实施战略性新兴产业发展三年行动计划，新型显示、高端制造、大数据等六大重点产业规模不断壮大，总投资280亿元的第6代AMOLED项目已试生产，达产后将满足9000万部智能手机全柔性折叠屏需求；总投资81亿元的中安信碳纤维及康得复材一期形成1700吨的年产能，总投资4.8亿元的河钢集团创新基地等一批重大项目签约落地，战略性新兴产业增加值占规上工业增加值比重达到22.7%，同比提高6.2个百分点。现代物流、文化旅游、电子商务等现代服务业加快发展，增加值同比增长14.3%，占服务业增加值比重达到65%。整体退出安次洸远钢厂，化解钢铁产能808万吨，妥善安置职工6000多人。开展“千企转型”行动，实施技改项目538项。设立1.5亿元财政专项奖励资金和“市长特别奖”，助推实体企业提档升级、增产增效。设立工业设计专项资金，建成全省首家市级工业设计创新中心。装备制造业增加值占规模以上工业增加值比重达到38.4%。实施科技创新三年行动计划，新增协同创新基地5家，总数达到27家；267家企业通过省高新技术企业专家评审，新增科技型中小企业1253家，新增市级以上孵化器和众创空间12家，新增院士工作站6家，万人发明专利拥有量达到4.85件；全市技术合同交易额达到88.5亿元，增长180%。我市入选中国城市创新竞争力百强。

(四)加快改革开放步伐，发展活力进一步释放。坚决落实中央和省各项改革部署，突出抓好投融资、科技、金融财税等九项改革。深入推进“放管服”改革，进一步提升审批效率，推出简单事项马上办、复杂事项加速办、应上尽上网上办、管家服务省心办“四办”服务承诺，277项行政许可事项全部实现网上运行，审批效率平均提高75%，市主城区学校建设项目审批时间由238个工作日压减至72个；加快推进商事制度改革，全面推行“五十证合一”，企业登记注册实行“审核合一、一人通办”和全程电子化，办照实现即来即办、立等可取，企业开办时间由20个工作日压减至4个，全市市场主体达到36.07万户，同比增长13.25%。全面落实减税降费政策，加快推进预算绩效管理改革。顺利完成市级机构改革，扎实推进县级机构改革。科教文卫、生态文明等领域改革稳步推进。积极开展“双创双服”活动，出台一系列支持民营经济发展的政策措施，建立健全政企面对面、政银企保对接等常态化企业帮扶机制，全市举办政企面对面对接帮扶活动102次，帮助企业解决问题431个，开展政银企保对接活动213次，帮助企业融资160亿元；开通政企直通服务网上平台，实现了政府与企业从线下到线上全方位的零距离互动对接。我市民营经济增加值占生产总值比重达到72%以上，上缴税金占全部税收比重达到90.7%，从业人员占比达到82.3%；新奥集团、华夏幸福、荣盛发展、宏泰发展等11家企业入选“河北省优秀民营企业百强”，大厂、永清、固安、霸州4个县(市)跻身“全省民营经济发展先进县十强”，我市成为唯一连续6次获得全省民营经济发展先进市的设区市。成功举办“5·18”经洽会、“9·26”农交会、首届雄安国际健康论坛、京津冀国际投洽会等重点展会80个，我市被评为“改革开放40周年中国优秀会展城市”，廊坊开发区入选“2018中国产业园区持续发展百强榜单”、获评“2018中国大数据明星产业园”。廊坊综合保税区获国务院正式批复，京东集团跨境电商项目建成投运。实际利用外资完成10.5亿美元，预计全年进出口完成465亿元，同比增长28.5%。区域合作扎实推进，“通武廊”协同发展12项合作工程顺利实施，“静沧廊”三地开展全方位战略合作。

(五)社会事业加快发展，民生保障进一步增强。全年财政民生支出505.6亿元，占一般公共预算支出的81.5%；20项民心工程全部完成。实施健康、助残、教育、产业就业、社保兜底、危房改造等系列攻坚行动，实现脱贫退出1474户、4189人，分别占到建档立卡总量的71.2%、79.6%；安次区被评为河北省脱贫攻坚奖先进集体。对口帮扶张家口5个深度贫困县，落实帮扶资金1.91亿元。进一步提高社会保障水平，城乡低保平均保障标准达到每人每月693元，城乡居民基础养老金最低标准达到118元，均为全省最高；城镇新增就业7.3万人，登记失业率继续保持全省最低；老旧小区改造完成114个，棚户区改造开工10741套，均超额完成省定任务。坚持优先发展教育，实施60所中小学、幼儿园新改扩建工程，补充教师2556名，创建标准化学校128所，顺利完成历时五年的“全面改薄”工程，全部县(市、区)通过“义务教育发展基本均衡县”国检验收。以文安、香河为试点启动实施基层卫生综合服务能力“双提升工程”，与首都儿研所、通州妇幼共建的廊坊儿童医院、妇产医院正式开诊，中国医学科学院北京肿瘤医院廊坊分院确定选址。成功举办中国图书馆年会、中超华夏幸福主场赛事，廊坊城市知名度、美誉度进一步提升。加快《廊坊市城市总体规划(2018—2035年)》编制；在主城区实施12条“断头路”区段打通工程，8个区段实现通车，其余区段正在按计划加快推进；精心谋划并开工建设光明道上跨京沪高铁立交桥项目，促进光明道东西更加畅通，主城区南北有机连接；外环路西南环线正式闭合通车，彻底结束外环路长期断环的历史；新建公厕129座，达到每平方公里4座的国家设置标准；认真整改市民服务中心遗留问题，一期工程具备入驻条件；殡仪馆迁建工程开工建设。积极开展农村人居环境治理，农村公路新改建完工里程突破500公里，清理积存垃圾1280万立方米，改造厕所7.47万座，香河县被评为“全省农村人居环境整治全域完成示范县”，大厂县被授予“全国文明城市提名城市”称号，固安县通过省级文明县城验收。深入开展安全生产隐患大排查、大整治攻坚行动，工矿商贸事故起数、死亡人数实现双下降，全省最低。认真做好军队退役人员管理服务工作，“两站两中心”建设、数据信息采集、悬挂光荣牌实现全覆盖。扎实推进防范化解重大风险、扫黑除恶专项斗争、治理拖欠农民工工资等工作，圆满完成全国两会、庆祝改革开放40周年大会等重大活动安保维稳任务，筑牢首都政治护城河。民族宗教、外事侨务、人民防空、史志档案、气象地震、地理信息、援藏援疆、妇女儿童等各项工作均取得了新成绩。

一年来，我们大力推进政府系统党的建设，坚决贯彻全面从严治党战略部署，扎实推进中央巡视、国务院大督查和省委巡视反馈问题整改，严格落实中央八项规定和实施细则精神，建立健全各项制度。不断加强法治政府建设，大力深化行政执法三项制度改革，严格执行重大行政决策合法性审查制度，顺利完成物业管理、广告设置等重点领域行政立法；自觉接受人大法律监督、工作监督和政协民主监督，重大事项向人大报告、向政协通报，人大代表建议144件、政协委员提案311件全部办复。

一年来，我们面对的形势纷繁复杂，工作中努力统筹兼顾。一是在经济下行压力明显加大的情况下，我们坚定不移落实国家和省关于规划、土地、环保以及房地产等各项管控政策，扎实推进“无钢市”“禁煤区”建设，有力服务保障了国家重大战略的顺利实施。二是在严格贯彻执行管控政策下，我们千方百计保增长，坚持稳中求进工作总基调，主要经济指标实现平稳运行，努力完成全年既定目标。三是在财政收入增速放缓的情况下，不遗余力惠民生，民生支出实现持续增长，占一般公共预算支出比例高于全省平均水平。四是在新旧动能转换的阵痛下，立足当前、着眼长远，针对基础设施、公共服务的短板弱项，做成了一些打基础、蓄后劲、利长远的事情，为我市高质量发展、可持续进步夯实了基础。

二、二○一九年工作总体要求和主要目标

今年政府工作的总体要求是：坚持以习近平新时代中国特色社会主义思想为指导，全面贯彻党的十九大、十九届二中、三中全会精神，深入落实中央经济工作会议、省委九届八次全会、省“两会”精神，按照市委六届六次全会战略部署，统筹推进“五位一体”总体布局，协调推进“四个全面”战略布局，坚持稳中求进工作总基调，坚持以新发展理念引领高质量发展，坚持以供给侧结构性改革为主线，坚持深化市场化改革、扩大高水平开放，深入贯彻“巩固、增强、提升、畅通”八字方针，全面落实“三六八九”工作思路，积极抢抓“五大发展机遇”，扎实推进“三大攻坚战”，紧紧围绕“四区一城”功能定位，全力做好“三篇大文章”，大力发展实体经济，推动民营经济高质量发展，狠抓重大项目建设，统筹做好科技创新、改革开放、乡村振兴、生态治理、民生保障、维护稳定各项工作，进一步稳就业、稳金融、稳外贸、稳外资、稳投资、稳预期，切实提高人民群众获得感、幸福感、安全感，保持经济持续健康发展和社会大局稳定，迈出新时代加快建设经济强市、美丽廊坊新步伐，为全面建成小康社会收官打下决定性基础，以优异成绩庆祝中华人民共和国成立70周年。

今年全市经济社会发展的主要预期目标是：地区生产总值增长6.5%左右，一般公共预算收入增长6%左右，固定资产投资增长6.5%左右，规模以上工业增加值增长3%左右，高新技术产业增加值增长8%左右，社会消费品零售总额增长10%左右，实际利用外资增长5%，城乡居民人均可支配收入分别增长7.5%和8%左右，城镇登记失业率控制在4%以内，全市PM2.5平均浓度、节能减排等指标完成省下达任务。

落实政府工作总体要求，实现今年发展预期目标，需要我们进一步提高政治站位，在市委的领导下，深入学习贯彻习近平新时代中国特色社会主义思想，树牢“四个意识”，坚定“四个自信”，做到“两个维护”，坚决贯彻习近平总书记对河北工作的重要指示，全面落实中央和省重大决策部署，推动我市经济社会发展始终沿着正确方向前进。需要我们进一步提高思想站位，强化机遇意识，增强辩证思维，正确看待挑战，变被动为主动，紧紧把握国家战略和国家大事进入全面实施的攻坚期，加快打造廊坊高质量发展的黄金期，特别是要充分看到，严管严控将是一种常态，习惯管控、适应管控必须成为一种自觉。积极克服消极等靠思想，主动摆脱传统路径依赖，加快改变固化思维模式，以等不及的紧迫感、慢不得的危机感、坐不住的责任感，逢山开路、遇水搭桥，凤凰涅槃、浴火重生，闯出一条高质量发展的新路来。需要我们进一步提高发展站位，牢牢抓住发展第一要务，自觉对标“国家大事、千年大计”，认真对表“世界眼光、国际标准、中国特色、高点定位”，切实把高质量发展作为解决一切问题的基础和关键，使劲儿向“雄安质量”看齐、向北京城市副中心标准靠近、向大兴国际机场速度追跑，正确处理好地方和全局、当前和长远、总量和结构、速度和质量的关系，把握好“稳、进、好、准、度”，持续推动经济社会稳步进入高质量发展的轨道。尤其是既要放眼全年，更要狠抓当前，把年度目标细化为季度、月度指标，分解到责任地、指定到责任人，狠抓生产要素保障，加强经济运行调度，以真抓的实劲、敢抓的狠劲、善抓的巧劲、常抓的韧劲，推动各项任务不折不扣落地落实、好于预期。需要我们进一步提高工作站位，始终坚持以人民为中心，把人民群众对美好生活的向往作为我们的奋斗目标。坚持做到对上对下负责相一致，把人民满不满意、高不高兴、答不答应，作为政府工作的出发点和落脚点。强化责任意识，激发担当精神，识大体、顾大局，敢负责、善作为，立足当前、着眼长远，只争朝夕、久久为功，善作善成、实干实政，切实当好首都政治护城河，努力成为全省高质量发展排头兵。

三、二○一九年重点工作任务

高标准抢抓协同发展三大历史性工程机遇，高水平统筹北中南三大板块协调联动，高质量做好北三县与通州区一体化发展、中部三区两县与大兴国际机场投入运营及临空经济区规划建设互联互动、南部一市两县与雄安新区规划建设对接衔接“三篇大文章”是今年重点任务的主题主线。

(一)高质量做好“三篇大文章”的规划编制篇。提高认识站位，持续加强与三大历史性工程对接规划体系，深入推进“五级两规一导则”建设完善，6月底前高质量完成规划期至2035年的城市总体规划和县(市)城乡总体规划。

加快完成北三县与通州区协同发展配套规划体系编制。加快完成临空经济区廊坊片区配套规划体系编制，统筹中部板块各项规划提升。加快南三县与雄安新区规划对接衔接。

(二)高质量做好“三篇大文章”交通建设篇。按照习近平总书记“把交通一体化作为推进京津冀协同发展的先行领域，加快构建三地快速、便捷、高效、安全、大容量、低成本的互联互通综合交通网络”要求，全面服务保障北中南三大板块综合交通体系建设，加快实现与三大历史性工程交通一体化。

在与北京通州区交通互联方面。积极推进京唐铁路(廊坊段)建设，全力保障项目建设顺利实施；主动配合做好平谷线前期工作，力争年内开工。加强与北京沟通对接，力促燕潮大桥对接开通，启动燕郊西出口拓宽改造工程；加快京沪高速北延、大厂厂通路潮白河公路新桥、香河安石路西延等公路项目前期工作，争取早日开工建设。

在与大兴国际机场及临空经济区交通互联方面。加快推进机场北线高速公路廊坊段和空港新区连接线建设，力争与北京东延段同步建成使用；全面保障城际铁路联络线一期工程(廊坊段)建设，力争天津至大兴国际机场联络线尽快开工；加快大兴国际机场与临空经济区、市主城区之间路网及临空经济区内路网建设，打造快捷的综合交通网络。

在与雄安新区交通互联方面。继续推进京雄城际铁路、津石高速(廊坊段)建设；积极配合推进京港台高铁丰雄段和京雄高速、荣乌高速新线、京德高速前期工作，加快津雄城际、雄安新区至大兴国际机场快线以及S327省道、S325省道等5条干线公路项目的对接研究。

(三)高质量做好“三篇大文章”的产业发展篇。着力加强重点产业、重点项目建设，进一步扩大对外开放，推动传统产业转型升级，为构建现代化经济体系提供坚实支撑。

大力发展新兴产业。深入实施战略性新兴产业发展三年行动计划，着力提升“大智移云”产业首位度，加快壮大新型显示、新材料新能源、高端制造、电子商务、大数据、文化创意六大千亿级产业集群，打造高端高新产业聚集区。各县(市、区)进一步明确产业定位和发展方向。

加快推进重点项目建设。深入开展“高质量项目建设年”活动，实施总投资920亿元的30个省重点项目、总投资1175亿元的100个市重点项目和800个亿元以上项目建设。持续强化市县两级领导包联重点项目制度，进一步完善项目建设调度、观摩、问责等机制，狠抓项目履约率、开工率、投资完成率，推动重点项目高质量建设。

积极扩大高水平对外开放。全力提升开发区发展能级，加快14个省级以上开发区基础设施建设向“新九通一平”迈进，切实增强对高端项目的吸引力、承载力；完善考核评价和动态管理机制。加强开发区精准开发建设。

加快推动传统产业转型升级。坚决退出文安新钢全部产能，实现“无钢市”目标。深入实施“千企转型”行动。大力提升农业现代化水平。优化房地产市场供给结构。

(四)高质量做好“三篇大文章”的创新驱动篇。深入落实科技创新三年行动计划，依托石保廊全面创新改革试验区、京南国家科技成果转移转化示范区，积极引进京津科技创新资源，加快形成科技成果“京津研发、廊坊孵化转化产业化”新格局。

培育壮大创新主体。精准培育高新技术企业，打造一批排头兵企业，鼓励中小企业创新发展，扩大科技型中小企业规模，进一步健全需求为导向、企业为主体的产学研用一体化创新机制，促进企业、高校、科研院所等创新资源有效整合。

加强创新载体建设。继续实施孵化器和众创空间三年倍增计划，大力支持创新载体提质增效，加速域内外技术、人才、资金等创新要素集聚，形成一批产业化成果；争取更多研发平台进入省级、国家级建设序列。

增强创新支撑能力。深入落实科技创新系列支持政策，建立市县财政科技资金快速增长机制，发挥各类创新创业引导基金作用，大力支持科技创新。推进人才兴廊工程，制定实施更具灵活性和吸引力的人才政策。

(五)高质量做好“三篇大文章”的生态保护篇。深入贯彻习近平生态文明思想，扎实做好中央和省环保督察问题整改，强化与京津雄生态环境联建联防联治，全力建设京津走廊生态宜居城市。

深入推进区域大气环境治理。严格执行《打赢蓝天保卫战三年行动计划》，进一步完善工作机制和考核奖惩制度，狠抓基层网格化建设与监测信息综合应用，继续六大行动，确保年度目标如期完成。严格落实“一企一策”，科学实施差异化管控，禁止“一刀切”式停限产。进一步加强与京津雄在污染源治理、重污染天气应急响应等方面的协调联动，积极开展信息共享、联合执法，共同推动空气质量持续改善。

坚决打赢水污染治理攻坚战。深入开展流域综合治理、水源地保护、城镇污水和黑臭水体治理、工业污水达标整治工作，重视中水利用，实施生态调水，全力改善水生态环境。

扎实推进生态修复保护。以创建国家森林城市为目标，进一步加大与京津雄交界区域造林力度，着力打造零梯度对接、无差异连贯的生态过渡带，使全市绿化规模、档次、水平再上新台阶。继续实施涉酸企业排查整治，全力推进垃圾处理处置、固体废物污染防治、农村环境综合整治三大行动。

(六)高质量做好“三篇大文章”基础设施篇。着眼于更好地融入京津冀协同发展大格局和以首都为核心的世界级城市群建设，按照“四区一城”功能定位，坚持大临空建设理念，强化国际门户意识，深入实施“基础设施和公共服务设施建设年”活动，进一步提升城乡综合承载能力。特别是切实增强主城区及临空经济区的凌空形象和俯瞰魅力。

实施一批城市标志性工程。实施一批旧城改造工程。实施一批城市断头路打通工程。实施一批城市生态景观工程。实施一批城市“里子”工程。实施一批城市垃圾处理工程。实施一批城市配套设施工程。实施一批农村基础设施工程。

(七)高质量做好“三篇大文章”的服务保障篇。深化“双创双服”活动，持续优化营商环境，切实强化政务保障、商务保障、社会保障，进一步增强服务发展、服务民生能力，加快推动我市实现高质量发展。

继续深化“放管服”改革。积极做好证明事项告知承诺制度全国改革试点工作。进一步深化商事制度改革，开展企业开办提速、证照分离专项行动，推进“多证合一”扩容升级。全面实施市场准入负面清单制度，确保民营企业依法平等进入负面清单以外各类行业和领域。加强事中事后监管，推进跨部门“双随机”抽查全覆盖，抽查结果公示率达到100%。深化工程建设项目审批制度改革。大力推进“互联网+政务服务”，做实“最多跑一次”“一次办好”等便民服务，最大限度便企利民。同时，统筹做好金融财税、科教文卫等领域改革。

全力支持民营经济发展。大力开展“服务民营企业高质量发展年”活动，集中实施对话企业家、千名干部入企服务等十项行动，切实为企业排忧解难。要不折不扣兑现落实省市支持民营经济发展的政策措施。坚决落实减税降费政策。积极构建亲清新型政商关系。

着力保障和改善民生。持续加大民生投入，实实在在为群众解难题、增福祉，实施20项民心工程。坚决兜住民生底线。全力办好人民满意的教育。着力满足群众多层次就医需求。推进文化体育强市建设。

全力保障社会安全稳定。狠抓安全生产工作，深入开展隐患排查整治，坚决遏制各类生产安全事故发生。加强食品药品安全监管，保障舌尖上的安全。推进“平安廊坊”建设。坚决打好防范化解重大风险攻坚战。深入开展扫黑除恶专项斗争。

加强国防教育和国防后备力量建设，做好退役军人服务保障工作，维护军人军属合法权益，让军人成为全社会尊崇的职业。充分发挥工会、共青团、妇联等人民团体桥梁纽带作用。做好民族宗教、外事侨务、人民防空、史志档案、气象地震、援藏援疆、妇女儿童、老龄、残疾人等工作。

忠诚履职尽责 以实绩实效让人民群众满意

为人民服务、对人民负责、受人民监督、让人民满意，是政府必须坚守的信念；增强落实能力、提升施政水平、推进高质量发展，是政府自身建设的永恒主题。我们一定不忘初心、牢记使命，锤炼过硬本领，忠诚履职尽责，以实绩实效让人民群众满意。我们要把坚持党的领导作为政府工作的定海神针，进一步树牢“四个意识”、坚定“四个自信”、做到“两个维护”，坚决贯彻落实中央和省各项决策部署，按照市委工作要求，奋发有为推进政府工作。我们要把为民服务作为政府工作的出发点和落脚点，始终把人民放在心中最高位置，扑下身子干实事、真心实意解民忧，使人民群众的获得感、幸福感、安全感更加充实、更有保障、更可持续。我们要把依法行政作为政府工作的根本遵循，忠实履行宪法和法律赋予的职责，依法行政、依法办事，自觉接受人大法律监督、政协民主监督、社会舆论监督，让人民监督权力，让权力在阳光下运行。我们要把实干实政作为政府工作的基本要求，不断增强“八种本领”，切实做到“五个过硬”，以钉钉子的精神、功成不必在我的境界和功成必定有我的担当，确保各项工作落地落实。我们要把廉洁持政作为政府工作的重要保障，坚决落实全面从严治党主体责任和党风廉政建设责任制，严格执行中央八项规定及其实施细则精神，驰而不息反“四风”，努力打造忠诚干净担当的高素质公务员队伍。
